# Omitting the Eradication Phase of Treatment of Adults with Melioidosis: A Pilot, Single-Center Randomized Controlled Trial

**DOI:** 10.1093/ofid/ofag591

**Published:** 2026-09-28

**Authors:** Simon Smith, James Stewart, Enzo Binotto, Jennifer Ho, Trent Yarwood, Valerie Nancarrow, Julian Harris, John Wood, Thomas Ragh, Christopher Reilly, Emily Shiel, Elizabeth Catchpoole, Josh Hanson

**Affiliations:** Department of Medicine, Cairns Hospital, Cairns, Queensland, Australia; Department of Medicine, Cairns Hospital, Cairns, Queensland, Australia; Department of Medicine, Cairns Hospital, Cairns, Queensland, Australia; Department of Medicine, Cairns Hospital, Cairns, Queensland, Australia; James Cook University, Cairns, Queensland, Australia; Department of Medicine, Cairns Hospital, Cairns, Queensland, Australia; James Cook University, Cairns, Queensland, Australia; Rural Clinical School, University of Queensland, Brisbane, Queensland, Australia; Department of Medicine, Cairns Hospital, Cairns, Queensland, Australia; Department of Medicine, Cairns Hospital, Cairns, Queensland, Australia; Department of Medicine, Cairns Hospital, Cairns, Queensland, Australia; James Cook University, Cairns, Queensland, Australia; Department of Pharmacy, Cairns Hospital, Cairns, Queensland, Australia; Department of Pharmacy, Cairns Hospital, Cairns, Queensland, Australia; Department of Medicine, Cairns Hospital, Cairns, Queensland, Australia; Department of Medicine, Cairns Hospital, Cairns, Queensland, Australia; Department of Medicine, Cairns Hospital, Cairns, Queensland, Australia; Kirby Institute, University of New South Wales, Sydney, New South Wales, Australia

**Keywords:** adverse reactions, Burkholderia pseudomallei, disease management, drug therapy, drug-related side effects, melioidosis

## Abstract

**Background:**

Contemporary Australian guidelines for the management of melioidosis recommend a longer duration of intravenous antibiotics during the intensive phase of treatment; recurrent disease is now far less common. A high rate of adverse drug reactions (ADRs) to the currently recommended minimum of 3 months of oral trimethoprim/sulfamethoxazole (TMP/SMX) eradication therapy questions the incremental value of this phase of treatment.

**Methods:**

In this pilot, single-center, open-label randomized controlled trial, adults with culture-confirmed melioidosis received either prolonged intravenous intensive-phase therapy followed by oral eradication-phase therapy with TMP/SMX (standard therapy) or prolonged intravenous intensive-phase therapy alone (short-course therapy). The primary outcome was culture-confirmed recurrence at 2 years. Secondary outcomes included clinical (culture-unconfirmed) recurrence and ADRs to TMP/SMX.

**Results:**

Among 33 participants, 5 had culture-confirmed recurrence: 0/16 in the standard therapy group and 5/17 (29%) in the short-course therapy group. One participant (6%) in the standard therapy group had clinical recurrence. An ADR to TMP/SMX occurred in 13/16 (81%) in the standard therapy group, necessitating TMP/SMX cessation in 7/13 (44%), hospitalization in 5/13 (38%), and intensive care unit admission in 1/13 (8%).

**Conclusions:**

Prolonged intravenous antibiotics alone will cure many patients with melioidosis; however, some still require oral eradication therapy. The potential for recurrence of life-threatening melioidosis must be balanced against the high rate of significant ADRs to high-dose TMP/SMX. This necessitates a greater understanding of the individuals at highest risk of recurrence and determination of the optimal dosing and duration of eradication therapy in those who need it.

## Teaser

Prolonged intravenous antibiotics alone will cure many patients with melioidosis, however some still require oral eradication therapy. The potential for recurrence of life-threatening melioidosis must be balanced against the high rate of significant adverse drug reactions to high-dose trimethoprim/sulfamethoxazole.

Melioidosis, caused by the environmental, opportunistic, gram-negative bacterium *Burkholderia pseudomallei,* is endemic to Northern Australia [1, 2]. Most antibody-generating exposures to *B*. *pseudomallei* are asymptomatic, but infection can develop and may be rapidly fatal, particularly in people with predisposing comorbidities that increase the likelihood of severe disease [2, 3]. Pneumonia is the most common presentation, bacteremia occurs frequently, and multi-organ involvement is often present, with the liver, spleen, prostate, skin, central nervous system, bones, and joints all potential sites of infection [4].

The management of melioidosis is complex; *B. pseudomallei* is intrinsically resistant to multiple classes of antibiotics, which limits therapeutic options, and surgery is often required for deep-seated abscesses [5]. Furthermore, the duration of antibiotic therapy for melioidosis—determined by the clinical phenotype and response to therapy—is prolonged and occurs in 2 phases, an intensive phase of intravenous antibiotics followed by an eradication phase of oral antibiotics, which starts immediately after completion of the intensive phase [6, 7]. In Australia, intravenous meropenem or ceftazidime is given for a period of 2–8 weeks during the intensive phase, with high-dose trimethoprim/sulfamethoxazole (TMP/SMX), the preferred oral agent for the eradication phase, given for 3–6 months [7].

However, even with effective antibiotics, melioidosis can recur, either as a recrudescent infection (defined as recurrence of culture-confirmed infection during the eradication phase, regardless of whether oral antibiotics are taken or not) or as a relapsed infection (defined as recurrence of culture-confirmed infection occurring after completion of the eradication phase), which occurs most frequently within 1 year after ceasing intravenous therapy [7–12]. In contemporary Australian series, the risk of melioidosis recurring in people who survive, who complete their intensive phase, and who are prescribed their oral eradication phase is ∼6%–7% [4, 11].

The rationale for a prolonged eradication phase of antibiotics is based, in part, on a 2006 Thai study of 2517 patients with culture-confirmed melioidosis [13]. Of these patients, 1229 (49%) died during hospitalization, and relapse occurred in 86/889 (9.7%) with follow-up data. The duration and choice of oral antimicrobial therapy were the most important determinants of relapse, with individuals receiving 12–16 weeks of oral therapy having a 90% decreased risk of relapse compared with individuals receiving ≤8 weeks [13]. However, in this study, the duration of intensive-phase antibiotics was frequently brief and less than in current Australian practice, with 59% of patients receiving ≤14 days of intravenous therapy [13]. Limited access to the sophisticated imaging and surgical support necessary to achieve optimal source control may also have contributed to an increased likelihood of relapse.

The intravenous antibiotics used in the treatment of melioidosis are usually well tolerated, but when high-dose TMP/SMX is used for the oral eradication phase, 51% of patients will have an adverse drug reaction (ADR) and 44% will require cessation or dose reduction [14]. These adverse reactions may be mild—such as gastrointestinal disturbance and rash—but more serious events, including acute kidney injury, bone marrow suppression, and drug rash with eosinophilia and systemic symptoms (DRESS), can occur [15, 16]. Some side effects, including Stevens Johnson Syndrome and toxic epidermal necrolysis, may even be fatal [17]. These adverse reactions, coupled with the recognition that there is often poor adherence to TMP/SMX during the oral eradication phase, have informed guidelines developed in the Northern Territory (NT) of Australia, which now recommend a prolonged intensive phase of intravenous antibiotic therapy [18]. This approach has resulted in low rates of disease recurrence, even in those who take only some—or even none—of their prescribed oral eradication therapy. In a study of 215 patients from the NT, only 108 (50%) completed their guideline-directed eradication therapy, but only 11 (5.1%) recrudesced and only 1 (0.5%) had a relapsed infection. Furthermore, 52/58 (90%) patients in this series who took no eradication therapy at all were cured; 2 (3.4%) died during the eradication phase, 3 (5.2%) recrudesced, and 1 (1.7%) relapsed. The study found that eradication therapy adherence did not predict recrudescence or relapse, which were instead more commonly explained by self-discharge from medical care, underlying immunosuppression, or an infected foreign body [7].

We therefore conducted the Short Course Antibiotic for Melioidosis Treatment (SCATMAN) pilot trial to determine if prolonged intensive-phase antibiotics alone (short-course therapy) would result in a similar rate of recurrence compared with prolonged intensive-phase antibiotics plus eradication-phase antibiotics (standard therapy) in adults with melioidosis.

## METHODS

### Study Design

This investigator-initiated, open-label randomized controlled trial was conducted at Cairns Hospital in Northern Australia. Adults aged ≥18 years were recruited between April 6, 2022, and June 13, 2025. The study was approved by the Metro North Human Research Ethics Committee (HREC/2021/QRBW/79751), and written informed consent was obtained from each participant. The study was prospectively registered at the Australian and New Zealand Clinical Trials Registry (ACTRN12622000225718).

### Participants

Participants were eligible for the study if they met the following inclusion criteria: (1) *B. pseudomallei* cultured from a clinical specimen; (2) able to be randomized during the intensive phase of treatment. We excluded participants meeting any of the following criteria: (1) suspected or confirmed central nervous system infection, bone or joint infection, or endovascular infection; (2) an expectation of death during the intensive phase with or without treatment; (3) previous participation in the trial; (4) a concurrent medical condition requiring TMP/SMX prophylaxis; (5) a concurrent medical condition requiring long-term treatment with TMP/SMX, doxycycline, or amoxicillin/clavulanate; (6) pregnancy; (7) unwillingness of the treating clinician to enroll the participant into the trial due to concerns about safety or the reliability of follow-up.

### Interventions

Randomization was balanced in permuted blocks of 20, with treatment allocation kept in sequentially numbered, sealed envelopes. Eligible participants were randomized 1:1 to receive either standard therapy (intensive-phase antibiotics [intravenous meropenem or ceftazidime], adjusted for renal function and weight, administered for 14–42 days depending on the antibiotic duration determining focus as decided by the treating clinician, followed by oral TMP/SMX adjusted for renal function and weight and prescribed for 90 days) or short-course therapy (intensive-phase antibiotics [intravenous meropenem or ceftazidime], adjusted for renal function and weight, administered for 14–42 days depending on the antibiotic duration determining focus as decided by the treating clinician), with no oral eradication phase [19].

In both groups, the duration of intensive-phase therapy was timed from the latest positive *B. pseudomallei* culture until the completion of intravenous antibiotics. Clinicians were encouraged to follow the revised Darwin melioidosis guidelines, which act as the Australian national guidelines, when deciding the dose and duration of intensive-phase antibiotics [11, 18]. The addition of TMP/SMX during the intensive phase was permitted, although this is not routine practice at our center, with the exception of introducing TMP/SMX in the last week of the intensive phase to facilitate the identification of early ADRs.

### Outcomes

The primary outcome was recrudescence or relapse of culture-confirmed melioidosis within 2 years of the initial culture-confirmed diagnosis of melioidosis. Multilocus sequence typing of isolates was used to confirm recrudescence or relapse rather than reinfection. Secondary outcomes were (1) all-cause mortality at 30 days and 90 days; (2) clinician suspected—but culture-unconfirmed—recurrence of melioidosis; (3) the proportion of participants with a medication-related adverse event at the end of intensive-phase antibiotics; (4) the proportion of participants with a medication-related adverse event at the end of eradication-phase antibiotics.

Participants were reviewed weekly for 12 weeks following completion of their intensive-phase antibiotics to assess for medication adherence, medication adverse events (if allocated to the standard therapy group), and symptoms suggesting recrudescence. Participants had blood collected for routine analyses at 4, 8, and 12 weeks following completion of their intensive-phase antibiotics, with more frequent blood tests if clinically indicated. Participants were reviewed at 6, 12, and 24 months for evidence of relapse. A safety card with instructions on how to contact a study investigator during the study period was provided to all participants.

### Adverse Events

All adverse events related to either meropenem or ceftazidime during the intensive phase and to TMP/SMX during the intensive phase (if commenced) and the eradication phase were recorded. This included any peripherally inserted central catheter–related complication, any new rash including DRESS or other severe cutaneous adverse reaction, gastrointestinal disturbance, bone marrow suppression (any new hemoglobin <135 g/L, neutrophils <2.0 ×10^9^/L, platelets <150 ×10^9^/L), acute kidney injury (increased creatinine ≥2× from baseline), hyperkalemia (serum potassium ≥5.5 mmol/L), or liver function test derangement (liver function tests ≥2× the upper limit of normal). The threshold for a severe adverse event that required cessation of TMP/SMX was determined by the treating clinician.

### Statistical Analysis

We estimated that the primary outcome would occur in 6% of participants in both groups and estimated that an adverse reaction to TMP/SMX requiring cessation of the drug would occur in 30% of participants in the standard therapy group. Assuming a 2-sided alpha of .05 and 80% power, this required a sample size of 28 participants per group; allowing for dropouts, we calculated a sample size of 60 participants. Data were analyzed on an intention-to-treat basis using statistical software (Stata, version 18.5). Categorical variables were analyzed using the Fisher exact test. Time to recurrence was assessed using a Cox proportional hazards regression model.

### Study Oversight

Study oversight was provided by an independent data and safety monitoring board (DSMB) comprised of an independent statistician and 2 independent infectious disease physicians with expertise in treating melioidosis. A planned interim analysis occurred after the first 30 participants had completed their initial 12-week follow-up. At this analysis in August 2025, the DSMB noted a substantial difference in recurrence rate between the 2 study groups and recommended ceasing or pausing study recruitment based on predetermined stopping guidelines (Supplementary Data). The principal investigators reviewed this recommendation and ceased recruitment on August 19, 2025. At the time of manuscript submission (August 7, 2026), 21/33 (64%) participants had completed 24 months of follow-up and/or met the primary end point, and the remaining 12 (36%) participants had all completed >13 months of follow-up.

## RESULTS

During the study period, there were 274 patients with culture-confirmed melioidosis; 33 were enrolled in the study (Figure 1, Table 1). The baseline characteristics of the 126 patients excluded due to clinician decision or who declined to participate are presented in Supplementary Table 1. In the 33 participants, the median (interquartile range [IQR]) duration of intensive-phase therapy was 28 (28–29) days in the standard therapy group and 28 (28–29) in the short-course therapy group. All durations of intensive-phase therapy were consistent with the Darwin melioidosis guidelines +/−3 days; 1 participant in the short-course therapy group received 41 days for management of a large liver abscess after repeat imaging at 4 weeks revealed ongoing peripheral enhancement. There was no difference in medication-related adverse events during intensive-phase therapy between the standard-therapy group and the short-course therapy group (9/16 [56%] vs 7/17 [41%]; *P* = .49). (Supplementary Table 2). No participant had to change or cease intensive-phase therapy.

**Figure 1.**
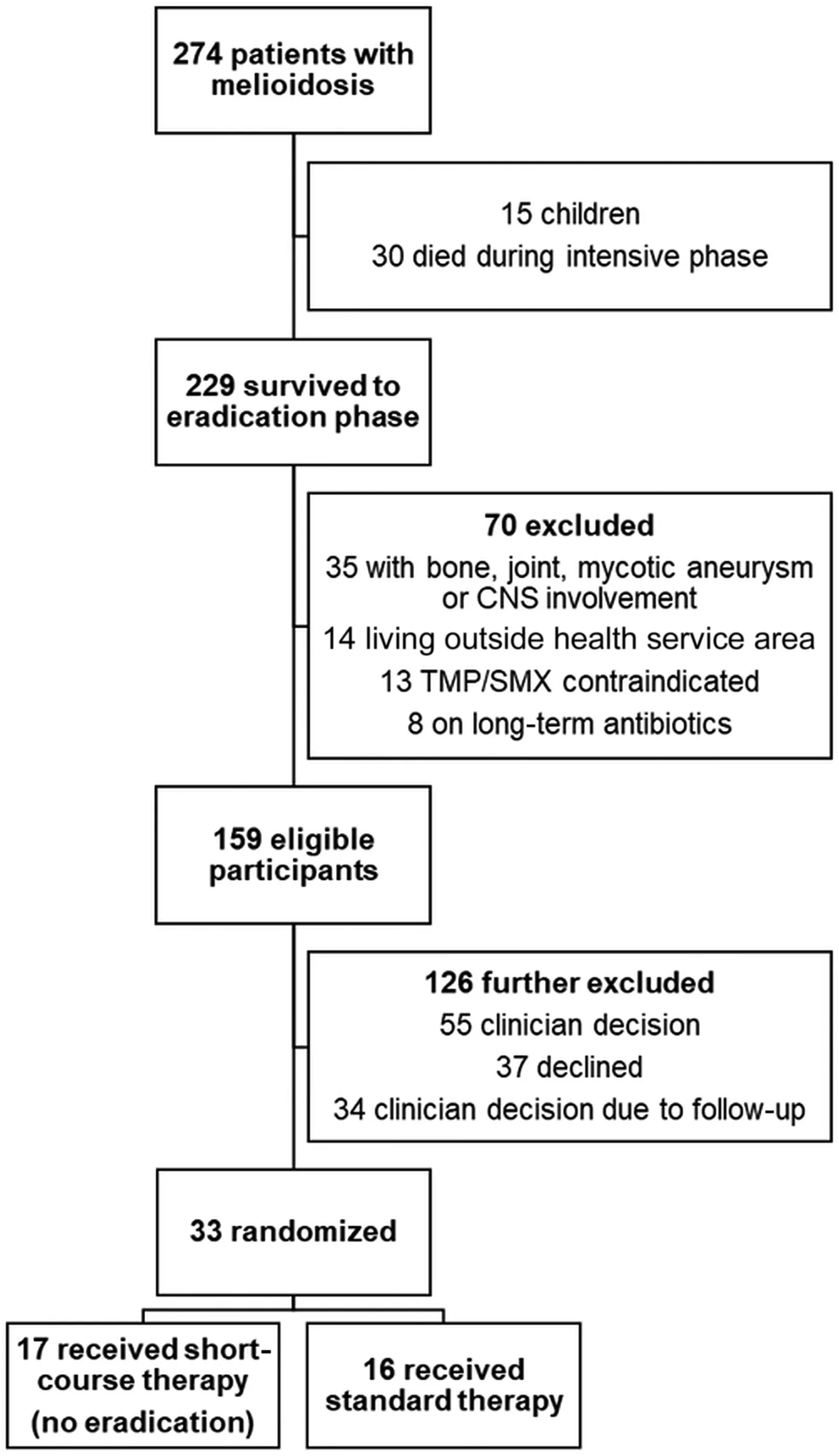
Consort diagram of participant recruitment into the study. Abbreviations: CNS, central nervous system; TMP/SMX, trimethoprim/sulfamethoxazole.

**Table 1.** Baseline Characteristics of Participants

Characteristic	Short-Course Arm (No Eradication)	Standard-Course Arm	Total
Randomized participants			
Participants randomized, No.	17	16	33
Participants randomized but who withdrew consent	0	0	0
Participants randomized but lost to follow-up	0	0	0
Age at randomization, median (IQR), y	49 (42–64)	67 (48–71)	60 (46–68)
Sex, No. (%)			
Male	11 (65)	10 (63)	21 (64)
Female	6 (35)	6 (38)	12 (36)
Identify as Aboriginal or Torres Strait Islander Australian, No. (%)	9 (53)	6 (38)	15 (45)
Nature of presentation, No. (%)			
Acute	17 (100)	12 (75)	29 (88)
Chronic	0	4 (25)	4 (12)
Comorbidities, No. (%)			
Diabetes mellitus	11 (65)	9 (56)	20 (61)
Hazardous alcohol use	5 (29)	4 (25)	9 (27)
Chronic kidney disease	0	0	0
Chronic lung disease	6 (35)	7 (44)	13 (39)
Malignancy	0	0	0
Immunosuppression	1 (7)	1 (7)	2 (7)
Clinical manifestation that led to the patient′s presentation, No. (%)			
Pneumonia	15 (88)	12 (75)	27 (82)
Bacteremia	1 (6)	0	1 (3)
Cutaneous involvement	0	4 (25)	4 (12)
Genitourinary involvement	1 (6)	0	1 (3)
Organ involvement, No. (%)			
Pulmonary	16 (94)	15 (94)	31 (94)
Bacteremia	8 (47)	10 (63)	18 (55)
Liver	3 (18)	0	3 (9)
Spleen	2 (12)	0	2 (6)
Genitourinary	3 (18)	2 (13)	5 (15)
Cutaneous	1 (6)	4 (25)	5 (15)
Myositis	1 (6)	1 (6)	2 (6)
Deep abscess present, No. (%)	7 (41)	3 (19)	10 (30)
Drainage procedure, No. (%)			
Prostate	3 (18)	2 (13)	5 (15)
Liver	1 (6)	0	1 (3)
Spleen	0	0	0
Muscle	0	1 (6)	1 (3)
Pulmonary empyema	1 (6)	1 (6)	2 (6)
Skin	1 (6)	1 (6)	2 (6)
Level of supportive care, No. (%)			
Intensive care unit admission	3 (18)	2 (13)	5 (15)
Intubation	0	0	0
Vasopressor support	2 (12)	1 (6)	3 (9)
Renal replacement therapy	0	0	0

Abbreviation: IQR, interquartile range.

Concurrent TMP/SMX was administered during the intensive phase in 12/16 (75%) in the standard therapy group; this was for ≤7 days in 11/12 (92%). One (6%) participant in the short-course therapy group received concurrent TMP/SMX during the intensive phase for 27 days; this was commenced due to persistent bacteremia for >14 days. Culture-confirmed recurrence occurred in 0/16 in the standard therapy group and in 5/17 (29%) in the short-course therapy group (Table 2). The median (range) time to recurrence was 43 (24–130) days from completion of intensive-phase therapy (Figure 2). There was 1 (6%) culture-unconfirmed recurrence in the standard therapy group, occurring in a 29-year-old man who re-presented 6 weeks post–intravenous therapy with multiple cerebral abscesses following treatment for pneumonia with renal and prostate abscesses. Cultures of blood and cerebrospinal fluid did not grow *B. pseudomallei*. There was no culture-unconfirmed recurrence in the short-course therapy group.

**Figure 2.**
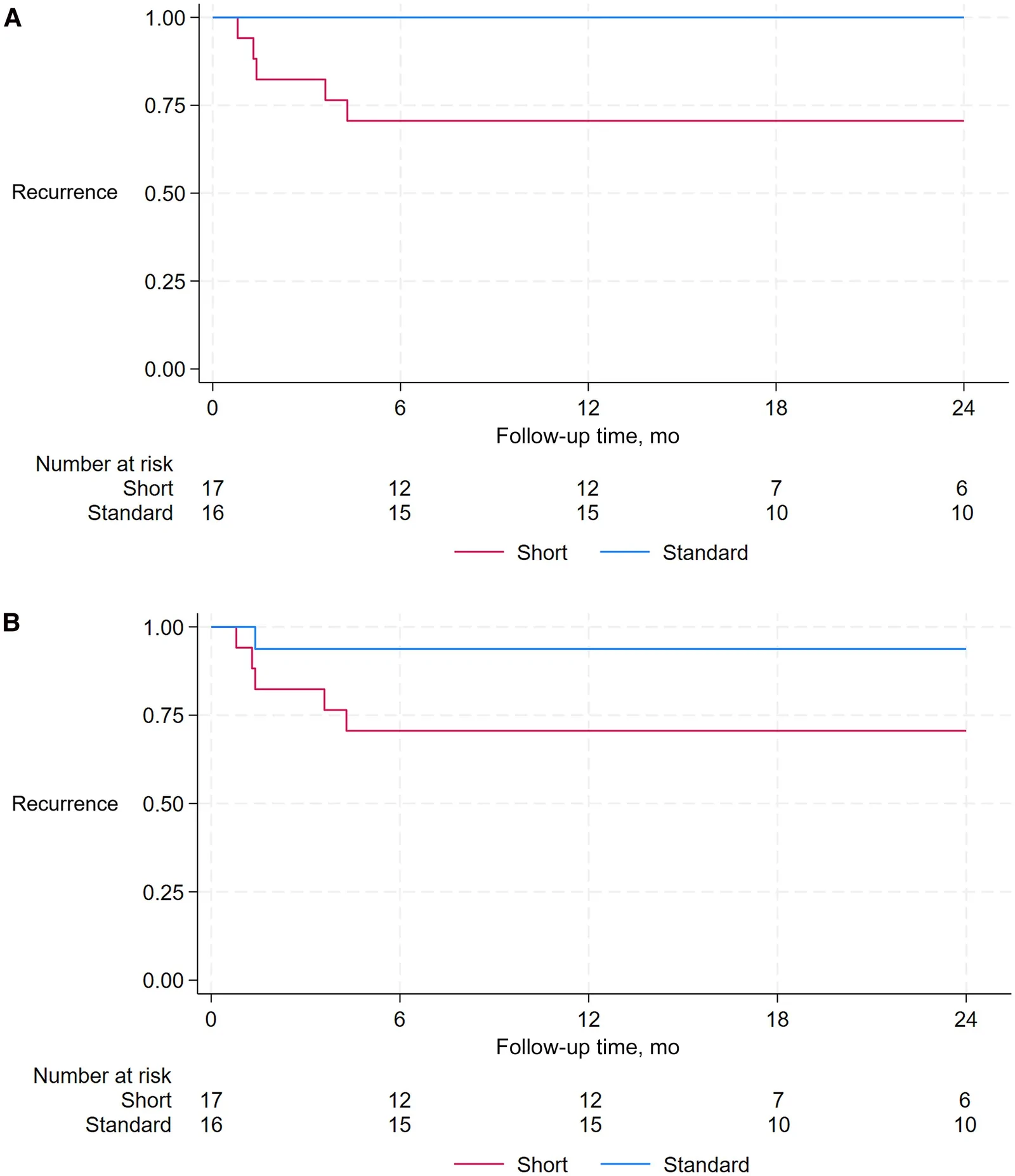
Time to culture-confirmed recurrence (*A*) and combined culture-confirmed and culture-unconfirmed recurrence (*B*) from completion of intensive-phase therapy.

**Table 2.** Clinical Characteristics and Treatment of Participants in the Short-Course Therapy Group who Had Culture-Confirmed Recurrent Melioidosis

Age/Sex	Risk Factor(s) for Melioidosis	Initial Clinical Manifestations	Bacteremic	Source Control	Duration of Intensive Phase, wk	Concurrent TMP/SMX, d	Time to Recurrence, d^a^	Recurrent Presentation
57 M	Chronic lung disease	Pneumonia, prostate abscess	No	TURP	4	0	43	Dysuria and frequency; no evidence of recurrent abscess on prostate MRI
44 F	Diabetes mellitus, hazardous alcohol	Pneumonia, skin abscess	No	Incision & drainage of skin abscess	3	0	130	Skin abscess
46 M	Hazardous alcohol	Pneumonia, prostate abscess	No	TURP	4	0	24	Testicular and groin pain; scrotal abscess requiring washout
29 M	Diabetes mellitus	Pneumonia, empyema, liver abscess, myositis	Yes	Thoracentesis	4	27	107	Left thigh myositis, right knee septic arthritis and femoral osteomyelitis^b^
65 F	Diabetes mellitus, chronic lung disease, immunosuppression	Pneumonia	Yes	N/A	4	0	39	Pneumonia

Abbreviations: MRI, magnetic resonance imaging; N/A, not applicable; TMP/SMX, trimethoprim/sulfamethoxazole; TURP, transurethral resection of the prostate.

^a^Timed from completion of intensive-phase therapy.

^b^Oral TMP/SMX commenced before culture confirmation due to clinical (culture-unconfirmed) concerns of relapse but subsequently culture-positive and restarted intensive phase.

In the standard therapy group, 13/16 (81%) had a TMP/SMX-related adverse event. This required cessation of TMP/SMX in 7/13 (54%) (Table 3; Supplementary Table 3), with hospitalization necessary for treatment of the adverse event in 5/13 (38%). Those hospitalized included 2 participants with an acute kidney injury and hyperkalemia, 1 participant with DRESS who required an intensive care unit admission, 1 participant with intractable nausea, and 1 participant who developed new renal calculi at the end of their eradication-phase therapy.

**Table 3.** TMP/SMX-Related Adverse Events That Necessitated Cessation of the Drug

Age/Sex	Adverse Drug Reaction	Abnormal Laboratory Results	Weeks Until Onset	Comments
38 F	DRESS^a^	Hemoglobin 77 g/L Neutrophils 1.2 ×10^9^/L	2	Fevers, tachycardia, hypoglycemia, generalized pain with pronounced edema and skin rash; required ICU admission for shock requiring vasopressor; recurrent infection excluded
82 M	Acute kidney injury	Creatinine 158 µmol/L^b^ Potassium 5.3 mmol/L	<1	Associated fatigue and confusion
70 M	Acute kidney injury, hyperkalemia	Creatinine 247 µmol/L^b^ Potassium 10.0 mmol/L	1	Associated fatigue—admitted to hospital for hyperkalemic emergency
72 M	Acute kidney injury, hyperkalemia	Creatinine 225 µmol/L Potassium 5.5 mmol/L	1	Known chronic renal impairment; serum creatinine usually ∼140 µmol/L
46 F	Intractable nausea, pruritis	Nil	1	Unable to eat and drink for 2 d due to intractable nausea
62 F	Acute kidney injury, hyperkalemia	Creatinine 220 µmol/L^b^ Potassium 5.5 mmol/L	3	Associated nausea; readmitted to hospital for management
84 M	Intractable nausea	Nil	1	Associated dizziness with falls; readmission for IV fluids

Abbreviations: DRESS, drug reaction with eosinophilia and systemic symptoms; ICU, intensive care unit; IV, intravenous.

^a^RegiSCAR 4 [20].

^b^Result ≥2× baseline creatinine.

Of the 12/17 (71%) participants allocated to short-course therapy who were cured, 7/12 (58%) were bacteremic and 4/12 (33%) had a deep-seated abscess (Table 4). All patients in the study were alive at 90 days.

**Table 4.** Clinical Characteristics and Treatment of Participants in the Short-Course Therapy Group who Were Cured After Intensive-Phase Antibiotics Alone

Age/Sex	Risk Factors	Clinical Manifestations	Bacteremic	ICU Admission	Source Control	Duration of Intensive Phase, wk	Concurrent TMP/SMX, d
24 F	Diabetes mellitus	Pneumonia, lymphadenopathy, liver abscess, splenic abscess	No	Yes	Liver abscess drainage	4	0
66 M	Chronic lung disease	Pneumonia, prostate abscess	No	No	TURP	4	0
35 F	Diabetes mellitus	Pneumonia, lymphadenopathy	No	No	N/A	4	0
62 M	Diabetes mellitus	Pneumonia, lymphadenopathy, pleural effusion	Yes	No	No	4	0
60 M	Diabetes mellitus, hazardous alcohol	Pneumonia	Yes	Yes	N/A	4	0
49 F	Diabetes mellitus, chronic lung disease	Pneumonia, splenic abscess	No	No	No	4	0
39 M	Diabetes mellitus	Pneumonia	No	No	N/A	3	0
69 M	Hazardous alcohol, chronic lung disease	Pneumonia with cavity	Yes	No	N/A	4	0
48 M	Hazardous alcohol	Pneumonia	Yes	No	N/A	4	0
70 F	Diabetes mellitus	Pneumonia	Yes	No	N/A	4	0
76 M	Chronic lung disease	Pneumonia	Yes	No	N/A	4	0
39 M	Diabetes mellitus	Pneumonia, liver abscess	Yes	No	No^a^	6	0

Abbreviations: ICU, intensive care unit; TMP/SMX, trimethoprim/sulfamethoxazole; TURP, transurethral resection of the prostate.

^a^Multiloculated liver abscess; not amenable to percutaneous drainage.

## DISCUSSION

This study suggests that oral eradication-phase therapy following intensive-phase intravenous antibiotics is still required by many people with melioidosis to reduce the risk of disease recurrence, even when prolonged intravenous antibiotic therapy is given. However, this must be weighed against the high rate of, sometimes life-threatening, adverse reactions to TMP/SMX.

The rate of recurrent disease in this study—29%—was much higher than observed in people who do not take prescribed oral eradication therapy in the NT. In the 5 participants who had a culture-confirmed recurrence in this study, 4 had recurrence at the same site despite initial thorough source control when this was possible. In the NT, clinicians usually coprescribe TMP/SMX in the intensive phase when managing neurological infection, osteomyelitis, septic arthritis, genitourinary infection, or skin and soft tissue infection [6, 7]. In our center, concurrent TMP/SMX is not routinely prescribed during intensive-phase therapy, and both the case-fatality rate and recurrence rate are historically very similar in both jurisdictions [2, 4].

The rationale for concurrent TMP/SMX during the acute phase of therapy is a theoretical improvement in antibiotic tissue penetration; however, there is no reduction in acute mortality in randomized controlled trials to support this approach [6, 21]. Additionally, a significant adverse reaction to TMP/SMX during the intensive phase often clouds management decisions, particularly when patients are concurrently receiving ceftazidime or meropenem, which may also be responsible for the reaction. It is possible that concurrent TMP/SMX during intensive-phase therapy reduces the risk of relapse, although further studies are required to confirm this. It is, however, notable that none of the 12 participants who were cured with short-course therapy in this study received concurrent TMP/SMX, including 4 (33%) who had a deep-seated collection.

Although the proportion of people who had recurrence in this study is much higher than the 6%–7% reported in contemporary Australian studies, it is still notable that >70% of participants achieved cure with intravenous antibiotics alone and were able to avoid potential side effects from oral eradication therapy [4, 11]. Indeed, an ADR to TMP/SMX occurred in >80% (and was life-threatening in 13%) of the participants in the standard therapy group, although this must be weighed against the risk of disease recurrence, which has been fatal in other series [2]. Ideally, TMP/SMX would be prescribed only to those at greatest risk of recurrence. However, identifying the highest risk patients reliably is likely to be challenging; in our study, recurrence occurred in 1 participant who only had pneumonia, while other participants with deep-seated abscesses were cured with short-course therapy.

Aside from ADRs, it is easy to appreciate why some patients with melioidosis do not take their prescribed eradication therapy. First, the duration of the eradication phase is long; patients must take therapy for at least 3 months, which can be challenging, even if there are no or only minimal side effects. Second, TMP/SMX tablets are large and difficult to swallow, particularly when “double strength” formulations are used. Third, melioidosis most commonly occurs in people with comorbidities who are frequently already taking multiple medications, and the addition of TMP/SMX (and folic acid) exacerbates this polypharmacy [2, 4]. Additionally, patients with hazardous alcohol use—the second most common risk factor for melioidosis in Australia—may also have difficulty adhering to prolonged courses of oral antibiotics [22].

Acknowledging these challenges, a minimum of 3 months of eradication therapy with TMP/SMX remains the treatment of choice for preventing a potentially life-threatening recurrence of melioidosis [23]. Current TMP/SMX dosing regimens are guided by a pharmacokinetic modeling study and excellent outcomes in large prospective observational studies [2, 24]. A large, multicentered randomized controlled trial that compared TMP/SMX with TMP/SMX plus doxycycline with culture-confirmed recurrent melioidosis as the primary end point found that TMP-SMX monotherapy was noninferior to TMP/SMX plus doxycycline and that patients taking TMP-SMX monotherapy had fewer ADRs [25]. While doxycycline monotherapy or amoxicillin/clavulanate may be used in people who cannot tolerate TMP/SMX—and they are both associated with fewer adverse reactions than TMP/SMX—these agents are less effective at preventing recurrence [23, 26, 27].

However, approaches to the antimicrobial therapy of melioidosis continue to evolve [23]. The ADRs seen in people taking TMP/SMX in the eradication phase of melioidosis are at least partly related to the high dose that is currently recommended, and they occur at a similar rate when TMP/SMX is used in higher doses for other conditions [28, 29]. In Australia, most *B. pseudomallei* isolates have a TMP/SMX minimum inhibitory concentration of ≤1 μg/mL [30], and effective concentrations may be able to be achieved with a lower dose of TMP/SMX, an approach that some authors have proposed [31], which is currently being explored in the LESSISMORE study (Australian and New Zealand Clinical Trials Registry ACTRN12625001247460). The role of novel oral agents including epetraborole, tebipenem, and ARV-1801 (ClinicalTrials.gov identifier: NCT05105035) requires further evaluation [32, 33]. Therapeutic drug monitoring might guide optimal dosing and enable further individualization of therapy, mitigating the risk of toxicity [34]. Meanwhile, prolonged intravenous therapy may be an alternative to TMP/SMX in select populations [35]. Other approaches, including optimizing the management of the comorbidities that predisposed individuals to developing melioidosis, might also be anticipated to reduce the risk of recurrence and improve general health outcomes. However, these strategies can be challenging to implement even in Australia's well-resourced and publicly funded health care setting [36, 37].

The limitations of this study include the small sample size; however, as recurrent melioidosis can been fatal, a feasibility study was essential before considering a larger trial. The results may still help support informed decision-making for people with melioidosis who are reluctant to commence—or at some point cease—TMP/SMX during planned eradication-phase therapy. The absence of placebo in the short-course therapy group may have overestimated adverse events attributable to TMP/SMX, although most participants in whom the medication was ceased had objective evidence to support this decision. Only 21% of eligible individuals with culture-confirmed disease during the study period were recruited to the study, with clinicians possibly unwilling to recruit participants perceived to have more complicated disease—and patients with central nervous system infection, bone or joint infection, or endovascular infection were excluded—limiting extrapolation to all people with melioidosis.

In conclusion, this pilot study provides data to support the use of eradication-phase oral therapy in melioidosis, while acknowledging that many people who receive longer courses of intensive intravenous therapy can achieve cure without this therapy. The high rate of adverse reactions to TMP/SMX underscores the need for larger prospective studies that define the optimal dosing and duration of oral TMP/SMX eradication treatment and identify the individuals who, in contemporary practice, are most likely to require this therapy.

## Supplementary Material

ofag591_Supplementary_Data

## Supplementary Data

Supplementary materials are available at *Open Forum Infectious Diseases* online. Consisting of data provided by the authors to benefit the reader, the posted materials are not copyedited and are the sole responsibility of the authors, so questions or comments should be addressed to the corresponding author.
