# Supplementary material for: Omitting the Eradication Phase of Treatment of Adults with Melioidosis: A Pilot, Single-Center Randomized Controlled Trial
Source: Open Forum Infect Dis. 2026 Sep 28;13(10):ofag591. doi: 10.1093/ofid/ofag591 (PMC13660701; doi:10.1093/ofid/ofag591)
Supplement: Supplementary file 1 [file ofag591_supplementary_data.docx]

**Supplementary**

**Supplementary Table 1.** Comparison of baseline characteristics of people with melioidosis included in the study and those who were excluded from the study due to clinician decision or who declined

| Characteristic | All n=159 | Included n=33 | Excluded n=126 | p |
| --- | --- | --- | --- | --- |
| Age (years, IQR) | 59 (44-69) | 60 (45-69) | 58 (44-70) | 0.62 |
| Male sex, n (%) | 105 (66) | 21 (64) | 84 (67) | 0.74 |
| First Nations Australians, n (%) | 69 (43) | 15 (46) | 54 (43) | 0.79 |
| Rural or remote residence, n (%) | 10 (6) | 2 (6) | 8 (6) | 1.0 |
| Wet season presentation, n (%) | 131 (82) | 24 (73) | 107 (85) | 0.12 |
| Acute presentation, n (%) | 141 (89) | 29 (88) | 112 (89) | 1.0 |
| Bacteremia, n (%) | 108 (68) | 18 (55) | 90 (71) | 0.06 |
| Diabetes mellitus, n (%) | 79 (50) | 20 (61) | 59 (47) | 0.16 |
| Hazardous alcohol use, n (%) | 50 (31) | 10 (30) | 40 (32) | 0.87 |
| Chronic lung disease, n (%) | 63 (40) | 13 (39) | 50 (40) | 0.98 |
| Chronic kidney disease, n (%) | 12 (8) | 0 | 12 (10) | 0.07 |
| Immunosuppression, n (%) | 12 (8) | 2 (6) | 10 (8) | 1.0 |
| Malignancy, n (%) | 19 (12) | 0 | 19 (15) | 0.01 |
| Pulmonary involvement, n (%) | 145 (91) | 31 (94) | 114 (90) | 0.74 |
| Musculoskeletal involvement, n (%) | 3 (2) | 2 (6) | 1 (1) | 0.11 |
| Skin and soft tissue infection, n (%) | 11 (7) | 6 (18) | 5 (4) | 0.01 |
| Genitourinary tract involvement, n (%) | 29 (18) | 5 (15) | 24 (19) | 0.80 |
| Visceral involvement, n (%) | 21 (13) | 4 (12) | 17 (13) | 1.0 |
| Abscess in any location, n (%) | 58 (36) | 14 (42) | 44 (35) | 0.43 |
| Septic shock, n (%) | 24 (15) | 4 (12) | 20 (16) | 0.79 |
| ICU admission, n (%) | 29 (18) | 5 (15) | 24 (19) | 0.80 |
| Vasopressor support, n (%) | 22 (14) | 3 (9) | 19 (15) | 0.57 |
| Mechanical ventilation, n (%) | 11 (7) | 0 | 11 (9) | 0.12 |
| Renal replacement therapy, n (%) ^a^ | 7/153 (5) | 0 | 7 (6) | 0.35 |
| Died before discharge, n (%) | 1 (1) | 0 | 1 (1) | 1.0 |

Abbreviations: IQR, Interquartile range

^a^ Excludes 6 already receiving long term hemodialysis

**Supplementary Table 2.** Medication-related adverse events occurring during the intensive phase, attributed to intravenous therapy and occurring prior to commencement of oral trimethoprim/sulfamethoxazole.

| Adverse event | Short course arm  (no eradication) n=17 | Standard course arm  n=16 | Total n=33 |
| --- | --- | --- | --- |
| Any, n (%) | 9 (53) | 7 (44) | 16 (48) |
| Elevated liver function tests, n (%) | 1 (6) | 4 (25) | 5 (15) |
| Nausea, vomiting or diarrhea, n (%) | 3 (18) | 1 (6) | 4 (12) |
| Rash, n (%) | 1 (6) | 0 | 1 (3) |
| PICC migration, n (%) | 1 (6) | 1 (6) | 2 (6) |
| Mucocutaneous candidiasis, n (%) | 1 (6) | 1 (6) | 2 (6) |
| Eosinophilia, n (%) | 2 (12) | 2 (13) | 4 () |
| Neutropenia, n (%) | 0 | 1 (6) | 1 (3) |

Abbreviations: PICC, peripherally inserted central catheter

**Supplementary Table 3**. Trimethoprim/sulfamethoxazole related adverse reactions that did not require cessation of the drug.

| Age/sex | Adverse drug reaction | Weeks until onset | Necessitating cessation of study drug | Comments |
| --- | --- | --- | --- | --- |
| 72 M | Gastrointestinal | 1 | No | Diarrhea when commencing TMP/SMX week 1 that resolved |
| 67 M | Gastrointestinal  Developed low density calculi in right renal pelvis and small stone right mid ureter | 1  12 | No  No | Nausea when commencing TMP/SMX week 1 that resolved  Stones evident on new CT at end of eradication phase, ureteric stent inserted but could not retrieve stone for analysis |
| 68 M | Gastrointestinal | 1 | No | Nausea when commencing TMP/SMX week 1 that resolved |
| 64 F | Gastrointestinal | 2 | No | Mild nausea only |
| 46 M | Gastrointestinal | 4-12 | No | Diarrhea that participant tolerated |
| 50 F | Gastrointestinal | 6-10 | No | Nausea and diarrhea that participant tolerated |
